# How Human Single-Neuron Recordings Can Help Us Understand Cognition: Insights from Memory Studies

**DOI:** 10.3390/brainsci11040443

**Published:** 2021-03-30

**Authors:** Zuzanna Roma Kubska, Jan Kamiński

**Affiliations:** Center of Excellence for Neural Plasticity and Brain Disorders: BRAINCITY, Nencki Institute of Experimental Biology, Polish Academy of Sciences, 02-093 Warsaw, Poland; j.kaminski@nencki.edu.pl

**Keywords:** human single-neuron recordings, cognition, concept cells, long-term memory, working memory, persistent activity

## Abstract

Understanding human cognition is a key goal of contemporary neuroscience. Due to the complexity of the human brain, animal studies and noninvasive techniques, however valuable, are incapable of providing us with a full understanding of human cognition. In the light of existing cognitive theories, we describe findings obtained thanks to human single-neuron recordings, including the discovery of concept cells and novelty-dependent cells, or activity patterns behind working memory, such as persistent activity. We propose future directions for studies using human single-neuron recordings and we discuss possible opportunities of investigating pathological brain.

## 1. Introduction

The way the human brain explores the world, acquires knowledge and processes information obtained through the senses has always been a mystery and a point of interest for philosophers and scientists alike. The last decades brought an abundance of research on cognitive processes, such as attention, memory, perception or executive functions, leading to many important and critical cognitive theories, some of which were divergent or even contradictory. Theories on working memory (WM) come to mind as an example. The widely known Baddeley and Hitch’s model divides WM into different, domain-specific (that is related to the sensory domain) components, while Cowan’s model focuses on attentional processes [1,2]. Although Cowan does not deny the existence of domain-specific stores in WM, he favors domain-general approach, especially when it comes to the maintenance mechanism of WM [3]. According to this approach, regardless of the sensory domain, neuronal networks maintain information using the same mechanism. Cowan also states that there are 4 items/chunks held in focus of attention, however in Oberauer’s model this number is limited to 1 [4]. Who, then, is right?

Although the question is simple, the answer is not. Cognitive neuroscientists have tried to address this and other questions, and figure out how the human brain works using different neuroimaging techniques, e.g., fMRI, EEG, MEG. These noninvasive techniques, however, come with many limitations. There is always a trade-off between spatial and time resolution [5]. EEG (or MEG) has very good time resolution, but provides limited insights about the locus of activity due to the inverse problem. fMRI, on the other hand, shows the locus of activity, but we can only infer about the neural activity from the blood-oxygen level dependent (BOLD) signal and, therefore, know little about the nature of neuronal activity: whether it is inhibitory or excitatory. Moreover, fMRI has poor time resolution [6,7,8].

## 2. Human Single-Neuron Recordings

Due to the limitations of noninvasive techniques and the fact that—despite an abundance of methods—little progress in solving the mystery of human cognition has been made to date [9], researchers resort to invasive techniques of recording brain activity, such as human single-neuron recordings. This technique has perfect spatial and time resolution, and although some limitations should be taken into account [10], it provides direct evidence of how the human brain works on cellular level and insights that would be impossible to obtain otherwise. Studies that led to the discovery of concept cells, that is cells that respond to the concept regardless of the way of presentation (auditory cues or different images), may serve as example [11,12]. What is even more important, researchers are able to use only verbal instructions and more advanced tasks than it is possible in studies on animals, which often require long days of training and use of simple tasks. We discuss concept cells and other discoveries further in this review, but for now we would like to stress that studies on humans using human single-neuron recordings gave us invaluable and otherwise unobtainable insights into our cognitive processes, such as memory, and the way the human brain processes information [10].

The possibility to record single neurons in humans comes from medical procedures in which implantation of the electrode into the human brain is a necessary part of the treatment. Currently, the bulk of research in this field comes from invasive epilepsy monitoring using the so-called “Behnke-Fried” electrodes [13]. Introduced in the late 1990s, these electrodes combine depth electrodes normally used for clinical purposes with microwires protruding from the end of the depth electrodes. Each electrode contains a set of eight microwires capable of recording single neurons. The placement of deep brain stimulation (DBS) electrodes in Parkinson’s or other diseases provides another opportunity to record neuronal activity in the human brain. In this procedure, neurosurgeons often use microelectrodes in order to improve the targeting of the DBS electrode, which makes it possible to record single-neuron activity along the trajectory of electrode implantation [14,15,16,17]. Research focusing on Brain Computer Interfaces (BCI), where electrodes implanted into the brain are being used, is yet another way we can record neuronal activity. Although the main goal of these projects is to create effective interfaces for paralyzed subjects, some of the experiments also deepen our knowledge of neuronal mechanism of cognitive system [18,19].

In all of the situations described above, using high impedance electrodes, we are able to record extracellularly action potentials of neurons in the near vicinity of the electrode. Next, using the procedure of spike sorting, we can extract the activity of putative neurons in human brain [20]. In the following paragraphs, we analyze some concepts formulated or confirmed thanks to this method, and analyze some of the conceptual frameworks of cognition from human single-neuron recordings perspective.

## 3. Grandmother, Gnostic and Concept Cells

Theories stating that relatively small groups of neurons, or—in extreme cases—even single neurons, code particular percepts in the brain date back to the 1960s, when two neuroscientists, Konorski and Lettvin, came up with two quite similar ideas almost simultaneously. Lettvin postulated that there are cells that respond to a picture of somebody familiar, e.g., the image of one’s grandmother, hence the name “grandmother cell” [21,22]. Konorski, on the other hand, proposed that there are cells (or cell assemblies) in the higher levels of cortical hierarchy that respond to complex stimuli and are responsible for coding object categories [23]. He called them “gnostic” cells. Both hypotheses were hard to verify experimentally until sparse, yet specific, responses of neurons in the medial temporal lobe (MTL) were discovered [12]. Thanks to human single-neuron recordings in epileptic patients implanted with depth electrodes, cells that responded to certain concepts were discovered. One of the first of such neurons was called the Jennifer Aniston neuron, as it responded selectively to different pictures of the actress Jennifer Aniston [12,24]. More specifically, it did not respond when the actress was depicted standing next to her former husband, Brad Pitt, yet responded to the image of her *Friends* costar Lisa Kudrow, even though Aniston was not present in the picture. This led to the conclusion that this particular neuron was responding not to the image of the actress herself, but probably to the concept of TV series “Friends”: as these two concepts (two actresses starring in the same series) were related semantically, they probably activated the same neuron [25]. Moreover, as De Falco and colleagues [25] demonstrated, such associations outstand task performance, therefore they are associated with neuronal mechanisms related to long-term memory. Diffused topography of responses enables associative learning and forming associations between distinct, visually unrelated concepts. The discovery was a step towards understanding neural mechanisms of abstract thoughts, something unique to humans. Similar results were found in a study where a cell that responded to Luke Skywalker also responded to the picture of Yoda, another “Star Wars” character [11]. Moreover, it was discovered that concept cells reacted selectively to certain visual stimuli even though they were linked only semantically and not visually (e.g., not only different pictures of a person, but also pencil sketches of them, or their name written down) [11]. That led to a conclusion that, unlike the grandmother or gnostic cells, these neurons fire to the semantic content and the concept of a person, not visual similarity. This finding was further supported by a study that showed that neurons responded to both visual and auditory stimuli referring to the same person (e.g., spoken or written names) [11]. In addition, it was discovered that the higher the level of hierarchical structure of MTL, the stronger the visual and multimodal invariance, which could mean that the higher level of organization, the higher the degree of abstraction [10]. The study also found a neuron that responded to both the Sydney Opera House and Bahai Temple in India (same patient whose other neuron responded to Jennifer Aniston). As it was later confirmed by the patient, he did not distinguish between these two buildings, which explains the firing of one neuron in response to two different stimuli. Note, that this insight would not be possible in animal studies, as feedback from the patient was crucial to the interpretation of this neural activity, which is a significant advantage of single-neuron recordings in human subjects.

These studies and another study by Quian Quiroga and colleagues [26] show that concept cells do not signal visual features of stimuli, but subjects’ perceptual decisions related to awareness. In the study mentioned [26] the subject was presented morphed, ambiguous pictures of two familiar faces (e.g., Bob Marley and Whoopi Goldberg), whereas a neuron that fired to one of them (Whoopi Goldberg) and not to the other (Bob Marley) was previously found. When the subject recognized Whoopi Goldberg’s morphed picture the stimulus-specific neuron responded strongly, which was not the case when the subject recognized Bob Marley. What is more, a linear classifier could predict the subject’s decision. Therefore, it is the decision and recognition (or categorization) of the stimulus that this MTL neuron fired to and not to visual features, and therefore this activity reflected consciousness. Moreover, research conducted by Reber and colleagues [27] using single-neuron recordings suggests gradual rather than all-or-nothing way of neural mechanisms of conscious perception as MTL stimulus-specific cells react to preferred stimulus even without conscious perception of the subject. What is even more important in this study, the higher up in the MTL hierarchy, the more neuronal response seems to be related to consciousness in terms of precise timing and magnitude, with anterior regions of MTL being the most prominent for conscious perception.

The discovery of concept cells is a chief example of the value of human single-neuron recordings. Those neurons were discovered in humans and most likely are unique to humans, as to date these specific cells were not found in other species [10], whose neurons seem to change their firing with contextual changes—unlike concept cells. The discovery of concept cells proved that at the end of the visual stream we can observe highly specialized cells which respond to high level features, as theorized in the previous century. Yet, cells recorded in MTL respond to visual stimulus as well as multimodal concepts. Those cells are also an invaluable tool for probing human cognition, allowing us to test conscious experience [26,27], organization of human knowledge [24,25], but also neuronal mechanisms of long-term memory or working memory [12,18,28,29,30], as explained towards the end of this review.

## 4. Insights from Human Single-Neuron Recordings to Long-Term Memory (LTM)

The role of MTL in memory processes is well documented. Observations of patients after lesions showed that this particular region plays a substantial role in forming new memories, successful consolidation and recall [31,32,33]. As the highest structure in the hierarchy of MTL’s organization, the hippocampus is involved in episodic memory [34,35] and association making [36]. As concept cells were discovered in MTL, they were considered in light of the role they can play in memory processes.

Our brain creates memories by making associations [37]. That is why we better remember things that are related to places we know and people we love. It is a well-known fact that it is easier to recall things in the place or circumstances they were learnt. One stimulus may trigger another if they were encoded together, are similar to each other, or our brain made an association/connection between them. This well observed effect was recently supported by a study that used human single-neuron recordings [28,38]. Once Ison and colleagues [28] found concept cells that responded to particular stimuli, they presented photoshopped pictures that contained these stimuli with a new one. For example, if a neuron originally responded to Jennifer Aniston and not the Eiffel Tower, the picture of the actress was photoshopped to show her standing near the Eiffel Tower. It turned out that after several expositions, the cell originally responding to Jennifer Aniston’s picture responded to the Eiffel Tower as well. Clearly, an association was made, and the neuron started to respond to both. This finding may mean that neurons in MTL play an important role in categorizing new information and integrating it with already existing concepts and memories. Possibly, concept cells are not units that form memory traces or stable representations of percepts per se, but are crucial for information encoding and consolidation processes. This view is consistent with studies on patients with lesions in MTL and their ability to remember old memories and difficulty in forming new ones [32,39]. Quian Quiroga, however, goes further with his interpretation and claims that categorization may happen in other brain areas and concept cells fire when conscious perception of stimulus occurs [24]. According to this theory, a concept cell brings stimulus into awareness, which enables association making and forming new memories in the flow of consciousness. Indeed, as described in the previous section, precise and stereotypical response of concept cells is associated with conscious experience [27].

**Figure 1:**
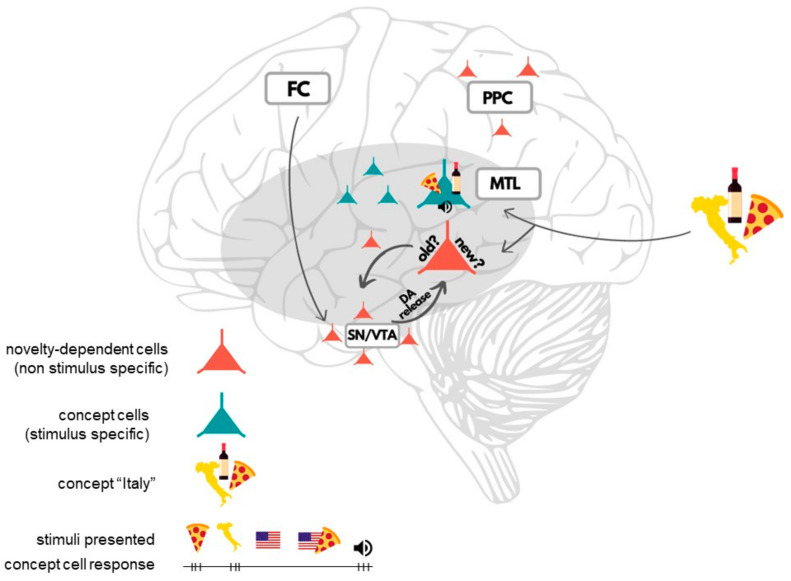
The figure shows two types of brain cells found using human single-neuron recordings: concept cells and novelty-dependent cells. Concept cells response in medial temporal lobe (MTL). Concept cells are activated by stimuli that form a part of the same concept and are associated semantically, but have no common visual features (e.g., concept: “Italy”; stimuli: country outline, pizza, wine), regardless of sensory modality, for instance an audio stimulus. The cell is not activated by stimuli if they form a part of a different concept (e.g., concept: “USA”) or are associated with stimuli that do not form a part of the “Italy” concept (e.g., pizza and the American flag) [24]. Novelty-dependent cells were found in the hippocampus, substantia nigra (SN) and posterior parietal cortex (PPC) [16,42,43,44]. Initial screening for novelty occurs in the hippocampus and it is further reinforced (or weakened) in SN and basal ganglia, depending on inputs from frontal cortex (FC) signaling top-down attention and one’s goals. Differentiating between the novelty vs. familiarity of a stimulus informs the brain if the stimulus is new and should be memorized. The functional hippocampal- SN/ventral tegmental area (VTA) loop and feedback dopamine release enhances LTP and enables successful memorization. Novelty-dependent memory-selective neurons in PPC play a role in memory-based choices.

Yet, concept cells are not the only finding that provided insights into cognitive processes thanks to human single-neuron recordings. Lisman and Grace proposed a theory of a functional loop between the hippocampus and substantia nigra/ventral tegmental area (SN/VTA) that is responsible for detecting novelty/familiarity and controlling the entry of information to LTM [40,41]. SN and VTA are key dopaminergic regions in the human midbrain. Firstly, novelty signals detected by the hippocampus are transferred to SN/VTA, where novelty-dependent cells are activated. Meanwhile, basal ganglia receive inputs from the prefrontal cortex (PFC) signaling top-down attention and motivation. Based on all the inputs, the enhancing/weakening of information occurs. Finally, feedback dopamine released to the hippocampus enhances long-term potentiation (LTP), which opens the entry to LTM and enables successful memorization [40,41]. Studies using human single-neuron recordings provided evidence in line with this theory. Researchers discovered populations of neurons in MTL that responded to the novelty vs. familiarity of stimulus with no regard to the category or visual features of the presented object [42,43,44] (Figure 1). Making a distinction between known and unknown information is crucial for memory. In other words, these neurons seem to “tell the brain” which stimuli are new and need to be memorized. What is interesting, similar neurons were found in the substantia nigra (SN) as well [16]. An analysis of action potentials and their characteristics led to the conclusion that these were probably dopaminergic neurons, and we already know dopamine plays a crucial role in cognitive processes such as attention and memory [40,41,45]. The study showed as well that the neurons that responded to novel stimuli in SN had longer response latency than similar cells in MTL. The difference in latencies may mean that after “the first screening for novelty” that occurs in the hippocampus, the information proceeds then to the basal ganglia (and SN), where it is further reinforced or weakened depending on one’s top-down attention and goals. These findings are coherent with Lisman’s and Grace’s concept of dynamic hippocampal-SN/VTA loop and its inputs from PFC. Moreover, Rutishauser and colleagues [18] using single-neuron recordings found two groups of neurons in the posterior parietal cortex (PPC) that signaled memory-based choices: a group of memory-selective novelty-dependent neurons (Figure 1) and confidence-selective neurons that differentiated between high- and low-confidence responses. The response of the former group of neurons was a function of confidence (subjective, regardless of truth), while the latter group signaled confidence independently of the novelty factor. This finding provides direct evidence for the involvement of PPC in declarative memory processes, such as transforming memories (knowledge) into choices based on confidence.

Humans have an exceptional ability to memorize new items. We can learn more than 2000 images after short exposition over a period of several days [46]. Studies using human single-neuron recordings described in this section helped to provide evidence for theoretical frameworks of this process, and revealed the nature of particular cells and their role in memory building: from categorization (concept cells) [24], to memorization process [16,42,43,44] (novelty-dependent neurons and dopamine secretion) to memory-based choices [18] (Figure 1).

## 5. Insights from Human Single-Neuron Recordings to Working Memory (WM)

As pointed out above, studies using human single-neuron recordings provided insights into the role of MTL in human long-term memory, showing the involvement of concept cells in association making and encoding, as well as finding neurons that distinguish between novel vs. familiar stimuli. Encoding new stimuli does not, however, occur only in long-term memory itself and is strictly related to WM and attention [2,4,47,48]. As suggested in Atkinson and Shiffirin’s model of long-term memory [47], working memory (called short-term storage) acts as a storage that transfers attended stimuli to long-term memory and receives input from LTM as well. WM is an important cognitive process that mediates other, more advanced cognitive functions, such as reasoning, calculation or problem solving [49]. Being so, human studies are irreplaceable as many of these processes are unique to human behavior and it is impossible to infer about them from animal studies. However, what can we learn about these aspects of cognition by analyzing human single-neuron recordings?

Animal studies set out the course for investigating human WM. Persistent activity (PA) recorded in animal brains made researchers suspect it could potentially be a mechanism for maintaining items in human WM [50,51]. Additionally, what is persistent activity? It occurs when neurons that responded to a stimulus during encoding keep on firing during the maintenance period when the stimulus is no longer present. It is supposed that the mechanism enables the brain to hold information in WM after a stimulus disappears. As human WM is far more complex than animal WM, scientists asked: is the same mechanism present in the human brain?

**Figure 2:**
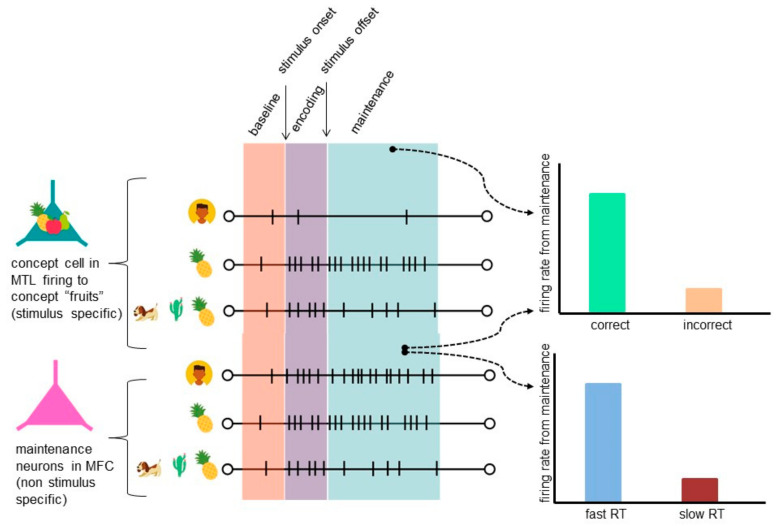
Persistent activity of concept cell (stimulus specific cell) in MTL and maintenance neurons (non-stimulus-specific cell) in medial frontal cortex (MFC) during working memory (WM) maintenance. A concept cell stays active during maintenance if preferred stimulus was present during encoding, yet activity decreases as a function of load. Persistent activity of the concept cell during maintenance correlates with the behavioral outcome (correctness) [29,30]. Persistent activity of a maintenance neuron decreases as a function of load. Its persistent activity correlates with the behavioral outcome (correctness and speed) [29].

Addressing the question became possible thanks to human single-neuron recordings. First evidence of persistent activity (PA) in human working memory was found by Kamiński and colleagues [29] almost simultaneously with Kornblith and colleagues [30] (Figure 2). Both studies were carried out on epileptic patients with depth electrodes implanted in MTL and both provided evidence that made it possible to consider persistent activity as a neural mechanism for WM. In both studies, neuronal activity during the maintenance period predicted behavioral outcome. Once Kamiński and colleagues identified concept cells, they showed that these cells continued their activity for a few seconds throughout the maintenance period after the stimulus offset. Similar neuronal activity was found in Kornblith and colleagues’ study, and although the number of neurons was small, their PA was highly significant and behaviorally relevant. This is an important finding as—with the number of neurons being small and their distribution sparse [24,52]—PA would probably be impossible to observe with noninvasive techniques and it is a unique insight from human single-neuron studies that would not be possible to obtain otherwise. Moreover, behavioral performance could be predicted based on the strength of stimulus-specific neuronal activity, which decreased as a function of working memory load [29].

These findings are in line with a view based on animal studies which states that PA is an important mechanism for WM [51,53,54]. They also provide first direct evidence for PA as a potential mechanism for WM maintenance in humans, however, at first they may seem incoherent with the fact that MTL is mainly considered to be a crucial structure for LTM. Although some patients with MTL lesions are as good as healthy controls when it comes to performing a WM task [32], their performance of more demanding tasks (longer maintenance time, higher load, or distractors) is impaired [55]. As WM seems to have a more distributed representation in the brain [56], a number of areas need to act in concert in order to perform WM tasks, especially the more demanding ones.

What is more, combining human single-neuron recordings with other methods provides unique insights. It also makes it possible to link specific single-unit activity with the activity of bigger neuronal populations from other regions and infer about their relation. For instance, Kamiński and colleagues [57] showed that the phase of slow oscillation in which a neuron fires can be used to read out content of WM (phase coding). In a different study Boran and colleagues [58] analyzed human single-neuron recordings from MTL together with iEEG in MTL and scalp EEG. They proved that persistent activity recorded in the hippocampus was involved in working memory processing, predicted workload during maintenance and showed that coupling between scalp and hippocampal EEG increased with higher workload. This finding supports the hypothesis about distributed organization of WM [56] and may lead to the conclusion that full WM capability is only possible when different regions connect and act in concert. (Which may explain why patients with LTM lesions perform well in simple WM tasks and show impaired WM functions in more demanding tasks, as mentioned earlier).

The precise capacity of WM remains an open question. Researchers cannot agree if the number is 7+/−2 [59], 4 as in Cowan’s model [60] or 1 as suggested by Oberauer [4], but studies based on human single-neuron recordings moved closer to finding the answer to that question. As shown, the frontal cortex (dACC and preSMA) is a region that contains maintenance neurons which show load-related activity and are independent of stimulus content unlike concept cells [29] (Figure 2). As the authors argue, since medial frontal cortex (MFC) lesions resulted in impaired switching between task sets [61], changes in load-dependent activity of these neurons and in RT may be due to different attentional demand and implementation of task sets, therefore these neurons may be related to WM’s central executive component, as they may inform what the current task requirements are (different at every stage of the task: encoding, maintenance or retrieval) [54]. Load-dependent neurons were found in the hippocampus as well [58], although finding these neurons in different brain areas may be due to different tasks and therefore different WM maintenance mechanisms involved (load-dependent vs. complex concept-dependent maintenance). If so, in order to estimate WM capacity, we may first need to determine these types of maintenance mechanisms.

To sum up, human WM is a complex cognitive process with distributed organization that differs a lot in its complexity as compared to animal WM. It gives rise to more advanced processes, such as reasoning or problem solving, which are unique to humans. Although invasive animal studies provide undoubtedly useful insights into neural correlates and the mechanisms of WM, they are not sufficient due to the complexity of human behavior. Animal studies with small loads, long hours of training and no feedback information from subjects do not shed light on the complexity of human cognition. Using human single-neuron recordings, we found direct evidence that non-stimulus-selective cells in human MFC are involved in successful WM performance and may be related to attentional control. We may also assume that similar cells in different regions of the brain may contribute to maintenance mechanisms, yet support different aspects of it.

## 6. How Human Single-Neuron Recordings Can Help Us Understand Cognition

**Figure 3:**
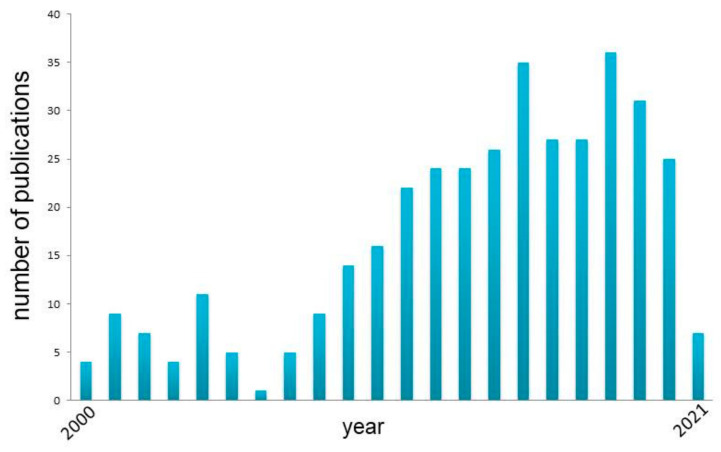
Increase in number of publications with phrase “human single-neuron recordings”. Data from Pub-Med.

There are many questions about neuronal mechanisms of human cognition that remain open. Due to technical limitations and ethical issues related to human single-neuron recordings, researchers cannot freely design cognitive studies. Therefore, it is important to carry out well designed studies that address questions which are basic, yet crucial for further scientific development. In this review, we described some major achievements on our quest to understand human cognition obtained via human single-neuron recordings. Over the last two decades, the number of studies using human single-neuron recordings almost doubled (Figure 3).

Nevertheless, this is just the starting point and many questions still await their answers. Future directions for studies using human single-neuron recordings may be considered from three different perspectives: (1) the kind of cognitive theories process investigated (and their aspects); (2) the location of electrodes (brain area); (3) the technology used.

The most important direction for cognitive studies using human single-neuron recordings are areas where the human mind is different from the mind of other mammals studied by neuroscience, such as the theory of mind, inference, language or working memory. Here, we will describe a few examples where this technique could help us to further our understanding of working memory.

Understanding the capacity of working memory, or the number of chunks of information we can hold in the focus of attention, will not only show us the capabilities of human mind, but will also support a given working memory model. As we mentioned earlier in this review, researchers cannot agree on the limits of WM capacity [4,59,60]. Many propose a different approach to the mechanism of working memory. Single-neuron recordings can make it possible to test WM capacity. For instance, researchers observed a decrease in stimulus-specific persistent activity with load [29] and no relation between the activity and position of the stimulus in the stream [30]. It is possible that when persistent activity is no longer distinguishable from the baseline activity load marks the capacity of working memory. Alternatively, we may be able to estimate WM capacity by counting cells that code individual stimuli in a unit of time determined by LFP oscillatory activity [62]. If we found that neurons which code different memoranda never fire together during WM task performance, we could assume that the number of information chunks in the focus of attention is limited to 1, as Oberauer predicted [4]. We might also look for differences in activity magnitude, assuming that the bigger the magnitude, the more attention is allocated to that memorandum and that memoranda with similar magnitude are probably at the same level of activation.

When it comes to WM maintenance, it also remains unclear whether the domain-specific or the domain-general approach is accurate [1,2,3]. However, we may address this question using human single-neuron recordings, by asking patients to memorize items represented in different modalities to test if those modalities are represented in persistent activity of the same cells. If so, it would be evidence in favor of the domain general approach; if not, it would mean that there actually are separate cellular storages for stimuli of different modality, like Baddeley and Hitch predicted. Preferably, while analyzing the outcomes, we should also bear in mind that different WM maintenance mechanisms may be involved (load- and complex concept-dependent maintenance) [29,58]. Maintaining stimuli in memory is one thing, but what is specific to human cognition is making operations on them. For years, the hypothesis of mental high-speed serial exhaustive scanning process (SES) was widely investigated and found confirmation in many studies (see [63]), but some researchers have their doubts, e.g., [64,65,66]. What is interesting in SES is that reaction time increases with load equally for both positive and negative trials. Supposedly, once a search for a probe is started, all items in the set are being scanned. It seems counterintuitive as the search should stop once an item is found and there is no need for further scanning. In order to check that on the cellular level, we could infer about SES existence from the activity of cells that code individual stimuli: cells’ response time positively correlated with load would be an argument in favor of SES.

The location of electrode implementation is predefined by surgical prerequisites, therefore, it cannot be manipulated freely. A vast majority of cognitive single-unit studies on humans are carried out on patients who require the implantation of depth electrodes for epilepsy monitoring or a DBS electrode to treat symptoms of Parkinson’s disease (PD) or essential tremor (ET). We have already obtained some insights into neuronal mechanisms of memory processes in MTL (concept cells, neurons for novelty/familiarity, etc.). Less human studies were performed using the recordings of single-unit activity in the frontal lobe [67], which is considered to be a crucial structure for our cognitive system. Here, the procedure of implantation of DBS electrodes makes it possible to record neuronal activity from the frontal cortex. As microelectrodes are often implanted as a part of standard procedure, recordings from this area do not pose additional risk to patients. This approach was already utilized by Ziv Williams group, showing that high quality neuronal recording can be obtained from the dorsolateral prefrontal cortex (dlPFC). This resulted in finding specific neurons that reflect subjective decisions [15] and neurons coding abstract rules [14]. The approach can, therefore, be applied in order to investigate cognitive processes at the single-neuronal level in PFC in order to obtain a broader view on cognition. Furthermore, we can record the activity of neurons in all structures along the trajectory of the DBS implantation. This includes several basal ganglia nuclei, such as the stratum or subthalamic nucleus. Besides motor functions, these can have an important role in cognition [68,69,70].

Treatment with DBS is promising and it is already applied or considered in many other diseases, like dystonia, obsessive-compulsive disorder, chronic pain, Alzheimer’s disease, Tourette syndrome, autism, or other psychiatric disorders (such as treatment-resistant depression, anorexia nervosa, mood disorders, addiction, schizophrenia, or anxiety disorders) [71,72]. Using DBS as a treatment in other diseases will provide us with access to many other brain areas, thus will create further research possibilities.

One of the aspects that holds back the research in the area of human single-neuron recording is slow development of electrode technology. For instance, the so-called “Behnke-Fried” electrode was introduced in the late 1990s and is still an accepted standard in single-neuron recording during epilepsy monitoring more than 20 years later [13]. This results from significant risks and effort associated with introducing new technologies in the difficult environment of human intracranial recording. In animal electrophysiology the main direction of electrode development took place in a silicone probe technology, but because of the fact that these electrodes are at risk of breaking, their usage in human brain is problematic. Still some new designs, either adapted from animal electrophysiology or designed specifically for humans, are emerging. NeuroGrid is one of the designs initially applied in animals, but successfully tested on humans [73,74]. These high-density, large-scale flexible microgrids are able to record action potentials from the surface of the cortex. Around of 840 mm^2^ of this grid can contain 240 electrodes, giving unprecedented recording capabilities in humans. The other electrode successfully applied in human electrophysiology is a linear probe [75,76]. Here, researchers embedded platinum-iridium contacts in polyimide-epoxy composite in order to increase structural rigidity. Thanks to multiple contacts, this electrode can record the activity of the whole human cortical column. Additionally, because the cortical column is a basic computational unit of the brain, this technology could be very useful in our endeavor to understand human cognition.

However, the electrodes described above are still only approved for single-case research use. One new technology already approved for clinical use are microelectrode arrays with sets of microwires implanted between ECoG electrodes [77]. Just like NeuroGrids, this electrode has capabilities to record single neuron activity from the cortex surface, although the number of contacts is much less impressive. Still, with this technology researchers were able to record grid cells in the entorhinal cortex [77]. Another new electrode close to being approved for humans combines a depth electrode used for epilepsy monitoring with microwire tetrodes [78]. In this design, tetrodes protrude from the main electrode for up to 2 mm. In animal research, tetrodes are commonly used, because they dramatically improve single-neuron isolation [79].

## 7. Human Single-Neuron Recordings of Pathological Brain

Human single-neuron recordings come with some limitations that we do not face using noninvasive techniques, such as EEG or fMRI. The number of study subjects is narrowed down to patients undergoing surgery due to brain pathology, e.g., [29,30,44]. What is more, brain coverage is limited as we can only implement electrodes in a few brain areas, not necessarily the ones of our scientific interest, which might be unaffected by the disease. However, possibly the greatest obstacle on our way to understanding human cognition is the fact that we always investigate the pathological brain and, therefore, potentially pathological activity.

Recording activity from the pathological brain may cast some doubts on the reliability of neuronal activity we record, however, awareness of limitations could help us overcome them and make reliable inferring. Since precise location of epileptic activity is unknown, electrodes in drug resistant epilepsy are implanted in different brain areas. Some of them are later classified as nonepileptic, which gives us access to undamaged tissue and we do not record pathological activity [42,80]. Comparing effects observed in healthy and pathological tissue is a useful tool to asses dependency of the results on epilepsy and in general revealed little difference [29]. Findings in line with earlier animal studies and evidence that confirms animal models give us reason to believe that recorded activity is unchanged by pathology. Moreover, thanks to the fact that epilepsy is not a homogenous disease [81], reproducible results from patients with different clinical pictures support the view that findings observed in epilepsy patients are not likely to be related to pathological changes in tissues, but rather represent physiological activity. As far as single-neuron recordings in Parkinson’s disease (PD) patients are concerned, researchers often record neurons from the substantia nigra (SN), which lies directly below the subthalamic nucleus, the common DBS target in Parkinson’s disease [16,17,82,83,84]. While SN suffers from massive loss of dopaminergic neurons in this disease, the loss is not uniform in the whole SN and the loss in dorsal areas is more comparable with that observed in same age healthy controls (unlike loss in the caudal part of SN pars compacta) [85]. As during the DBS implantation the dorsal parts of SN are most accessible, we are able to record activity from an area relatively untouched by disease. Furthermore we know from animal studies that the dopaminergic neurons have longer spike waveforms compared to GABAergic cells [86,87]. Neurons with longer spike waveforms were also observed in substantial nigra recordings in humans suggesting the possibility to record dopaminergic cells in PD [16,17]. Although we have to bear in mind that PD may influence the activity of the remaining neurons, no correlation has been observed between spike waveform length and PD stage [16]. Nevertheless, this issue remains open and requires further investigation.

Despite all their limitations, human single-neuron recordings create invaluable opportunities to record brain activity at its source and infer about direct neuronal mechanisms that underlie behavior. We choose to perceive investigating the pathological brain not as a limitation, but as an opportunity to gain knowledge on mechanisms behind pathology and be able to develop more accurate therapies, or even find early biomarkers of pathological activity.

Although cognitive impairment is not a feature hallmark in PD and ET diseases (motor impairment mainly is), a significant number of patients do show poorer cognitive performance than same age healthy controls. In PD and ET, we observe cognitive deficits in attention, memory, speed processing, and executive abilities [88,89,90,91]. Moreover, PD and ET patients face a greater risk of developing dementia than healthy controls [92,93]. It has been calculated that about 50% of PD patients develop MCI during the first 10 years after their diagnosis [94]. ET patients are also more likely to develop amnestic rather than nonamnestic MCI. A study suggests that the problem lies not only with the retrieval of information from memory but in the impairment of memory storage as such [95]. This shows that despite the above disorders being predominantly motor diseases, they must be associated with a factor or correlate that fosters cognitive impairment. The impairment profile is, however, not homogenous. Although many patients display a cognitive decline, some do not [93], and the cause of the disease is probably multifactorial [96], so defining what causes this cognitive impairment in some patients and not the others is hard at this point. This comes, however, as an opportunity to investigate the neuronal mechanism of cognitive functions and hopefully understand the underlying mechanisms and elaborate accurate, well addressed treatments drawing on the differences between cognitively impaired and unimpaired brains within one disease. Due to its disruptive effect on the quality of life, we can aim to study cognitive decline with single-neuron recordings. In the next paragraphs, we suggest some examples of possible future research directions.

Patients with temporal lobe epilepsy (TLE) also suffer from memory decline as well as widely reported deficits in recognition memory [97,98]. Some authors argue that there are two separate processes behind recognition memory: (1) recollection, where a memory is specifically retrieved via associations of details of an item or episode and (2) recognition based on familiarity, where not associations but “feeling of oldness” comes into play [97,98]. Indeed, several studies have shown that the former process is specifically disrupted in TLE [97,99]. Human single-neuron recordings may help us determine which of the processes is actually disrupted in TLE and where specific treatment should be targeted. Research using this recording technique already showed separate mechanisms in MTL that are responsible for recognition memory: concept cells that form blocks of our cognition and are a base for associative memory as well as specific cells that respond to novel vs. familiar stimuli regardless of stimulus features (both discussed above in this review) [24,42]. An analysis and comparison of the activity of these cells in epilepsy patients with TLE and with epilepsy localized in other brain regions could provide us with direct evidence of which mechanism of recognition memory is actually disrupted: recollection through associations or recognition based on familiarity, or maybe both.

Intracranial EEG studies which focus on pathological changes in TLE relate interictal epileptiform discharges (IED) with transient cognitive impairments [100,101,102]. Reed and colleagues [102] conducted a study using recognition tasks and correlated behavioral outcomes with activity of single neurons modulated by IED. When IED occurred just before stimulus onset, the recognition of familiar images was impaired. Such disruption was not present during recognition of novel stimuli. This finding supports the hypothesis that the process of recognition based on familiarity is disrupted in epilepsy patients. However, we still do not understand the exact mechanism behind this and future studies using human single-neuron recordings could bring new insights into the matter.

Similarly, to TLE, recognition and associative memory is also impaired in PD [103]. As we know from previous studies [16,42,43,44], neurons that respond to novelty vs. familiarity are not only observed in MTL, but were found as well in the basal ganglia, where they reinforce/weaken signals in the dynamic hippocampal-SN/VTA loop [40,41]. Neurodegeneration occurring in the basal ganglia (especially in the striatum and substantia nigra) may impair the functioning of these neurons and the hippocampal-SN/VTA loop, and thus cause a decline in recognition memory. It would be interesting to find out if a mechanism similar to TLE occurs in PD. If so, we may be able to find a common base of cognitive impairment in these motor diseases.

We believe that further studies using human single-neuron recordings may help us understand the neural basis of cognition. The fact that human single-neuron recordings can only be carried out on pathological brains, however limiting, comes as an invaluable opportunity to understand the mechanisms behind pathologies and lead to the development of well-targeted new treatments.

## 8. Conclusions

Single-neuron recordings are an invaluable tool in brain investigation. Using this technique, we can benefit from exceptional time and spatial resolution. So far, the majority of studies using single-neuron recordings were conducted on animals, which, however important, do not make it possible to grasp the complexity of human cognition. While animal studies serve as a signpost for human studies and can be both valid and insightful, the possibility of recording human brain using this technique is invaluable. Thanks to human single-neuron recordings, scientists managed to discover concept cells, which, to date, have not been found in other species and are probably unique to humans. We discovered mechanisms that underlie memory processes and were able to gain some insights into the neural basis of conscious perception and memory-based choices. Moreover, access to pathological human brain creates an opportunity to investigate the mechanisms behind pathologies and will, hopefully, enable the development of well-targeted therapies.

Last but not least, as intracranial implantation of electrodes carries a risk to the health of the patients, it is worth drawing attention to the safety of the procedure. The goal of understanding human cognition and designing well-targeted treatments for pathologies is important, yet electrode implantation always involves the risk of internal bleeding or damaging brain structures [104]. Therefore, there is a need to understand if implantation of research electrodes carries additional risks. To date, studies conducted to address this concern have showed that hybrid electrodes with microwires used for single-neuron activity recordings do not increase the risk of the procedure. They are as safe as standard electrodes [104,105] and combining them with intraoperative electrocorticography adds minimal risk to surgery [106,107].

## Data Availability

Data sharing not applicable.

## References

[1] Baddeley A., Hitch G. (1974). Working Memory. Psychol. Learn. Motiv..

[2] Cowan N., Elliott E.M., Saults S.J., Morey C.C., Mattox S., Hismjatullina A., Conway A.R.A. (2005). On the capacity of attention: Its estimation and its role in working memory and cognitive aptitudes. Cogn. Psychol..

[3] Li D., Christ S.E., Cowan N. (2014). Domain-general and domain-specific functional networks in working memory. Neuroimage.

[4] Oberauer K. (2002). Access to Information in Working Memory: Exploring the Focus of Attention. J. Exp. Psychol. Learn. Mem. Cogn..

[5] Burle B., Spieser L., Roger C., Casini L., Hasbroucq T., Vidal F. (2015). Spatial and temporal resolutions of EEG: Is it really black and white? A scalp current density view. Int. J. Psychophysiol..

[6] Bandettini P.A. (2014). Neuronal or hemodynamic? Grappling with the functional MRI signal. Brain Connect..

[7] Kim S.G., Ogawa S. (2012). Biophysical and physiological origins of blood oxygenation level-dependent fMRI signals. J. Cereb. Blood Flow Metab..

[8] Logothetis N.K. (2008). What we can do and what we cannot do with fMRI. Nature.

[9] Buzsáki G. (2019). The Brain from Inside Out.

[10] Quian Quiroga R. (2019). Plugging in to Human Memory: Advantages, Challenges, and Insights from Human Single-Neuron Recordings. Cell.

[11] Quian Quiroga R., Kraskov A., Koch C., Fried I. (2009). Explicit Encoding of Multimodal Percepts by Single Neurons in the Human Brain. Curr. Biol..

[12] Quian Quiroga R., Reddy L., Kreiman G., Koch C., Fried I. (2005). Invariant visual representation by single neurons in the human brain. Nature.

[13] Fried I., Wilson C.L., Maidment N.T., Engel J., Behnke E., Fields T.A., Macdonald K.A., Morrow J.W., Ackerson L. (1999). Cerebral microdialysis combined with single-neuron and electroencephalographic recording in neurosurgical patients: Technical note. J. Neurosurg..

[14] Mian M.K., Sheth S.A., Patel S.R., Spiliopoulos K., Eskandar E.N., Williams Z.M. (2014). Encoding of rules by neurons in the human dorsolateral prefrontal cortex. Cereb. Cortex.

[15] Jamali M., Grannan B., Haroush K., Moses Z.B., Eskandar E.N., Herrington T., Patel S., Williams Z.M. (2019). Dorsolateral prefrontal neurons mediate subjective decisions and their variation in humans. Nat. Neurosci..

[16] Kamiński J., Mamelak A.N., Birch K., Mosher C.P., Tagliati M., Rutishauser U. (2018). Novelty-Sensitive Dopaminergic Neurons in the Human Substantia Nigra Predict Success of Declarative Memory Formation. Curr. Biol..

[17] Zaghloul K.A., Blanco J.A., Weidemann C.T., McGill K., Jaggi J.L., Baltuch G.H., Kahana M.J. (2009). Human substantia nigra neurons encode unexpected financial rewards. Science.

[18] Rutishauser U., Aflalo T., Rosario E.R., Pouratian N., Andersen R.A. (2018). Single-Neuron Representation of Memory Strength and Recognition Confidence in Left Human Posterior Parietal Cortex. Neuron.

[19] Aflalo T., Zhang C.Y., Rosario E.R., Pouratian N., Orban G.A., Andersen R.A. (2020). A shared neural substrate for action verbs and observed actions in human posterior parietal cortex. Sci. Adv..

[20] Rey H.G., Pedreira C., Quian Quiroga R. (2015). Past, present and future of spike sorting techniques. Brain Res. Bull..

[21] Gross C.G. (2002). Genealogy of the “grandmother cell”. Neuroscientist.

[22] Quian Quiroga R., Fried I., Koch C. (2013). Brain cells for grandmother. Sci. Am..

[23] Konorski J. (1967). Integrative Activity of the Brain.

[24] Quian Quiroga R. (2012). Concept cells: The building blocks of declarative memory functions. Nat. Rev. Neurosci..

[25] De Falco E., Ison M.J., Fried I., Quian Quiroga R. (2016). Long-term coding of personal and universal associations underlying the memory web in the human brain. Nat. Commun..

[26] Quian Quiroga R., Kraskov A., Mormann F., Fried I., Koch C. (2014). Single-Cell Responses to Face Adaptation in the Human Medial Temporal Lobe. Neuron.

[27] Reber T.P., Faber J., Niediek J., Bostro J., Elger C.E., Mormann F., Elger C.E., Mormann F. (2017). Single-Neuron Correlates of Conscious Perception in the Human Medial Temporal Lobe Report Single-Neuron Correlates of Conscious Perception in the Human Medial Temporal Lobe. Curr. Biol..

[28] Ison M.J., Quian Quiroga R., Fried I. (2015). Rapid Encoding of New Memories by Individual Neurons in the Human Brain. Neuron.

[29] Kamiński J., Sullivan S., Chung J.M., Ross I.B., Mamelak A.N., Rutishauser U. (2017). Persistently active neurons in human medial frontal and medial temporal lobe support working memory. Nat. Neurosci..

[30] Kornblith S., Quian Quiroga R., Koch C., Fried I., Mormann F. (2017). Persistent Single-Neuron Activity during Working Memory in the Human Medial Temporal Lobe. Curr. Biol..

[31] Corkin S. (2002). What’s new with the amnesic patient H.M.?. Nat. Rev. Neurosci..

[32] Squire L.R., Stark C.E.L., Clark R.E. (2004). The medial temporal lobe. Annu. Rev. Neurosci..

[33] Squire L.R., Zola-Morgan S. (1991). The medial temporal lobe memory system. Science.

[34] Tulving E., Markowitsch H.J. (1998). Episodic and declarative memory: Role of the hippocampus. Hippocampus.

[35] Eichenbaum H., Yonelinas A.P., Ranganath C. (2007). The medial temporal lobe and recognition memory. Annu. Rev. Neurosci..

[36] Borders A.A., Aly M., Parks C.M., Yonelinas A.P. (2017). The hippocampus is particularly important for building associations across stimulus domains. Neuropsychologia.

[37] Brasted P.J., Bussey T.J., Murray E.A., Wise S.P. (2003). Role of the hippocampal system in associative learning beyond the spatial domain. Brain.

[38] Mayes A., Montaldi D., Migo E. (2007). Associative memory and the medial temporal lobes. Trends Cogn. Sci..

[39] Scoville W.B., Milner R.B. (1957). Loss of recent memory after bilateral hippocampal lesions. J. Neurol. Neurosurg. Psychiatry.

[40] Lisman J.E., Grace A.A. (2005). The hippocampal-VTA loop: Controlling the entry of information into long-term memory. Neuron.

[41] Lisman J., Grace A.A., Duzel E. (2011). A neoHebbian framework for episodic memory; role of dopamine-dependent late LTP. Trends Neurosci..

[42] Rutishauser U., Mamelak A.N., Schuman E.M. (2006). Single-trial learning of novel stimuli by individual neurons of the human hippocampus-amygdala complex. Neuron.

[43] Rutishauser U., Schuman E.M., Mamelak A.N. (2008). Activity of human hippocampal and amygdala neurons during retrieval of declarative memories. Proc. Natl. Acad. Sci. USA.

[44] Rutishauser U., Ross I.B., Mamelak A.N., Schuman E.M. (2010). Human memory strength is predicted by theta-frequency phase-locking of single neurons. Nature.

[45] Nieoullon A. (2002). Dopamine and the regulation of cognition and attention. Prog. Neurobiol..

[46] Standing L., Conezio J., Haber R.N. (1970). Perception and memory for pictures: Single-trial learning of 2500 visual stimuli. Psychon. Sci..

[47] Atkinson R.C., Shiffrin R.M. (1968). Human Memory: A proposed system and its control processes BT—The Psychology of Learning and Motivation. Psychol. Learn. Motiv..

[48] Oberauer K. (2019). Working Memory and Attention—A Conceptual Analysis and Review. J. Cogn..

[49] Baddeley A. (2012). Working memory: Theories, models, and controversies. Annu. Rev. Psychol..

[50] Fuster J.M., Alexander G.E. (1971). Neuron Activity related to short-term memory. Science.

[51] Constantinidis C., Funahashi S., Lee D., Murray J.D., Qi X.L., Wang M., Arnsten A.F.T. (2018). Persistent spiking activity underlies working memory. J. Neurosci..

[52] Olshausen B.A., Field D.J. (2004). Sparse coding of sensory inputs. Curr. Opin. Neurobiol..

[53] Compte A., Brunel N., Goldman-Rakic P.S., Wang X.J. (2000). Synaptic mechanisms and network dynamics underlying spatial working memory in a cortical network model. Cereb. Cortex.

[54] Kamiński J., Rutishauser U. (2020). Between persistently active and activity-silent frameworks: Novel vistas on the cellular basis of working memory. Ann. N. Y. Acad. Sci..

[55] Jeneson A., Squire L.R. (2012). Working memory, long-term memory, and medial temporal lobe function. Learn. Mem..

[56] Christophel T.B., Klink P.C., Spitzer B., Roelfsema P.R., Haynes J.D. (2017). The Distributed Nature of Working Memory. Trends Cogn. Sci..

[57] Kamiński J., Brzezicka A., Mamelak A.N., Rutishauser U. (2020). Combined Phase-Rate Coding by Persistently Active Neurons as a Mechanism for Maintaining Multiple Items in Working Memory in Humans. Neuron.

[58] Boran E., Fedele T., Klaver P., Hilfiker P., Stieglitz L., Grunwald T., Sarnthein J. (2019). Persistent hippocampal neural firing and hippocampal-cortical coupling predict verbal working memory load. Sci. Adv..

[59] Miller G.A. (1956). The Magical number 7 plus or minus two: Some limits on our capacity for processing information. Psychol. Rev..

[60] Cowan N. (2001). The magical number 4 in short-term memory: A reconsideration of mental storage capacity. Behav. Brain Sci..

[61] Gläscher J., Adolphs R., Damasio H., Bechara A., Rudrauf D., Calamia M., Paul L.K., Tranel D. (2012). Lesion mapping of cognitive control and value-based decision making in the prefrontal cortex. Proc. Natl. Acad. Sci. USA.

[62] Lisman J.E., Idiart M.A.P. (1995). Storage of 7 ± 2 short-term memories in oscillatory subcycles. Science.

[63] Sternberg S. (2016). In defence of high-speed memory scanning. Q. J. Exp. Psychol..

[64] Dosher B.A., Sperling G. (1998). A Century of Human Information-Processing Theory: Vision, Attention, and Memory.

[65] Henson R., Hartley T., Burgess N., Hitch G., Flude B. (2003). Selective interference with verbal short-term memory for serial order information: A new paradigm and tests of a timing-signal hypothesis. Q. J. Exp. Psychol. Sect. A Hum. Exp. Psychol..

[66] Jonides J., Lewis R.L., Nee D.E., Lustig C.A., Berman M.G., Moore K.S. (2008). The mind and brain of short-term memory. Annu. Rev. Psychol..

[67] Kawasaki H., Adolphs R., Oya H., Kovach C., Damasio H., Kaufman O., Howard M. (2005). Analysis of single-unit responses to emotional scenes in human ventromedial prefrontal cortex. J. Cogn. Neurosci..

[68] Weintraub D.B., Zaghloul K.A. (2013). The role of the subthalamic nucleus in cognition. Rev. Neurosci..

[69] Simpson E.H., Kellendonk C., Kandel E. (2010). A Possible Role for the Striatum in the Pathogenesis of the Cognitive Symptoms of Schizophrenia. Neuron.

[70] Hanganu A., Provost J.S., Monchi O. (2015). Neuroimaging studies of striatum in cognition part II: Parkinson’s disease. Front. Syst. Neurosci..

[71] Aum D.J., Tierney T.S. (2018). Deep brain stimulation: Foundations and future trends. Front. Biosci..

[72] Graat I., Figee M., Denys D. (2017). The application of deep brain stimulation in the treatment of psychiatric disorders. Int. Rev. Psychiatry.

[73] Khodagholy D., Gelinas J.N., Thesen T., Doyle W., Devinsky O., Malliaras G.G., Buzsáki G. (2015). NeuroGrid: Recording action potentials from the surface of the brain. Nat. Neurosci..

[74] Khodagholy D., Gelinas J.N., Zhao Z., Yeh M., Long M., Greenlee J.D., Doyle W., Devinsky O., Buzsáki G. (2016). Organic electronics for high-resolution electrocorticography of the human brain. Sci. Adv..

[75] Csercsa R., Dombovári B., Fabó D., Wittner L., Erss L., Entz L., Sólyom A., Rásonyi G., Szcs A., Kelemen A. (2010). Laminar analysis of slow wave activity in humans. Brain.

[76] Ulbert I., Halgren E., Heit G., Karmos G. (2001). Multiple microelectrode-recording system for human intracortical applications. J. Neurosci. Methods.

[77] Nadasdy Z., Nguyen T.P., Török Á., Shen J.Y., Briggs D.E., Modur P.N., Buchanan R.J. (2017). Context-dependent spatially periodic activity in the human entorhinal cortex. Proc. Natl. Acad. Sci. USA.

[78] Despouy E., Curot J., Reddy L., Nowak L.G., Deudon M., Sol J.C., Lotterie J.A., Denuelle M., Maziz A., Bergaud C. (2020). Recording local field potential and neuronal activity with tetrodes in epileptic patients. J. Neurosci. Methods.

[79] Buzsáki G. (2004). Large-scale recording of neuronal ensembles. Nat. Neurosci..

[80] Kamiński J., Rutishauser U. (2017). Insights on vision derived from studying human single neurons. Cogn. Sci. Technol..

[81] Steinlein O.K. (2008). Genetics and epilepsy. Dialogues Clin. Neurosci..

[82] Dayal V., Limousin P., Foltynie T. (2017). Subthalamic nucleus deep brain stimulation in Parkinson’s disease: The effect of varying stimulation parameters. J. Parkinsons Dis..

[83] Weiss D., Breit S., Wächter T., Plewnia C., Gharabaghi A., Krüger R. (2011). Combined stimulation of the substantia nigra pars reticulata and the subthalamic nucleus is effective in hypokinetic gait disturbance in Parkinson’s disease. J. Neurol..

[84] Mikell C.B., Sheehy J.P., Youngerman B.E., McGovern R.A., Wojtasiewicz T.J., Chan A.K., Pullman S.L., Yu Q., Goodman R.R., Schevon C.A. (2014). Features and timing of the response of single neurons to novelty in the substantia nigra. Brain Res..

[85] Damier P., Hirsch E.C., Agid Y., Graybiel A.M. (1999). The substantia nigra of the human brain: II. Patterns of loss of dopamine-containing neurons in Parkinson’s disease. Brain.

[86] Ungless M.A., Grace A.A. (2012). Are you or aren’t you? Challenges associated with physiologically identifying dopamine neurons. Trends Neurosci..

[87] Stauffer W.R., Lak A., Yang A., Borel M., Paulsen O., Boyden E.S., Schultz W. (2016). Dopamine Neuron-Specific Optogenetic Stimulation in Rhesus Macaques. Cell.

[88] Aarsland D., Creese B., Politis M., Chaudhuri K.R., Ffytche D.H., Weintraub D., Ballard C. (2017). Cognitive decline in Parkinson disease. Nat. Rev. Neurol..

[89] Barone P., Aarsland D., Burn D., Emre M., Kulisevsky J., Weintraub D. (2011). Cognitive impairment in nondemented Parkinson’s disease. Mov. Disord..

[90] Martinez-Horta S., Kulisevsky J. (2019). Mild cognitive impairment in Parkinson’s disease. J. Neural Transm..

[91] Prasad S., Shah A., Bhalsing K.S., Kumar K.J., Saini J., Ingalhalikar M., Pal P.K. (2019). Abnormal hippocampal subfields are associated with cognitive impairment in Essential Tremor. J. Neural Transm..

[92] Louis E.D., Joyce J.L., Cosentino S. (2019). Mind the gaps: What we don’t know about cognitive impairment in essential tremor. Park. Relat. Disord..

[93] Weil R.S., Costantini A.A., Schrag A.E. (2018). Mild Cognitive Impairment in Parkinson’s Disease—What Is It?. Curr. Neurol. Neurosci. Rep..

[94] Williams-Gray C.H., Mason S.L., Evans J.R., Foltynie T., Brayne C., Robbins T.W., Barker R.A. (2013). The CamPaIGN study of Parkinson’s disease: 10-year outlook in an incident population-based cohort. J. Neurol. Neurosurg. Psychiatry.

[95] Collins K., Rohl B., Morgan S., Huey E.D., Louis E.D., Cosentino S. (2017). Mild Cognitive Impairment Subtypes in a Cohort of Elderly Essential Tremor Cases. J. Int. Neuropsychol. Soc..

[96] Smith C.R., Cullen B., Sheridan M.P., Cavanagh J., Grosset K.A., Grosset D.G. (2020). Cognitive impairment in Parkinson’s disease is multifactorial: A neuropsychological study. Acta Neurol. Scand..

[97] Bowles B., Crupi C., Pigott S., Parrent A., Wiebe S., Janzen L., Köhler S. (2010). Double dissociation of selective recollection and familiarity impairments following two different surgical treatments for temporal-lobe epilepsy. Neuropsychologia.

[98] Illman N.A., Kemp S., Souchay C., Morris R.G., Moulin C.J.A. (2016). Assessing a Metacognitive Account of Associative Memory Impairments in Temporal Lobe Epilepsy. Epilepsy Res. Treat..

[99] Moscovitch D.A., McAndrews M.P. (2002). Material-specific deficits in “remembering” in patients with unilateral temporal lobe epilepsy and excisions. Neuropsychologia.

[100] Kleen J.K., Scott R.C., Holmes G.L., Roberts D.W., Rundle M.M., Testorf M., Lenck-Santini P.P., Jobst B.C. (2013). Hippocampal interictal epileptiform activity disrupts cognition in humans. Neurology.

[101] Horak P.C., Meisenhelter S., Song Y., Testorf M.E., Kahana M.J., Viles W.D., Bujarski K.A., Connolly A.C., Robbins A.A., Sperling M.R. (2017). Interictal epileptiform discharges impair word recall in multiple brain areas. Epilepsia.

[102] Reed C.M., Mosher C.P., Chandravadia N., Chung J.M., Mamelak A.N., Rutishauser U. (2020). Extent of single-neuron activity modulation by hippocampal interictal discharges predicts declarative memory disruption in humans. J. Neurosci..

[103] Das T., Hwang J.J., Poston K.L. (2019). Episodic Recognition Memory and the Hippocampus in Parkinson’s disease: A Review. Cortex.

[104] Carlson A.A., Rutishauser U., Mamelak A.N. (2018). Safety and utility of hybrid depth electrodes for seizure localization and single-unit neuronal recording. Stereotact. Funct. Neurosurg..

[105] Hefft S., Brandt A., Zwick S., Von Elverfeldt D., Mader I., Cordeiro J., Trippel M., Blumberg J., Schulze-Bonhage A. (2013). Safety of hybrid electrodes for single-neuron recordings in humans. Neurosurgery.

[106] Panov F., Levin E., De Hemptinne C., Swann N.C., Qasim S., Miocinovic S., Ostrem J.L., Starr P.A. (2017). Intraoperative electrocorticography for physiological research in movement disorders: Principles and experience in 200 cases. J. Neurosurg..

[107] Sisterson N.D., Carlson A.A., Rutishauser U., Mamelak A.N., Flagg M., Pouratian N., Salimpour Y., Anderson W.S., Richardson R.M. (2021). Electrocorticography During Deep Brain Stimulation Surgery: Safety Experience From 4 Centers Within the National Institute of Neurological Disorders and Stroke Research Opportunities in Human Consortium. Neurosurgery.

